# Multicomponent coordination-driven self-assembled metallacycles as efficient photosensitizers for photodynamic therapy

**DOI:** 10.1016/j.jpha.2026.101644

**Published:** 2026-04-27

**Authors:** Jinping Zhang, Yali Hou, Qian Feng, Dengke Han, Qi Sun, Yuling Wu, Sida Liu, Dong Liu, Fei Xue, Jin Zhang, Jianing Wang, Xianglong Duan, Feng Liu, Mingming Zhang

**Affiliations:** a Second Department of General Surgery, Shaanxi Provincial People’s Hospital, Xi’an, 710068, China; b Shaanxi Engineering Research Center of Medical Polymer Materials, Xi’an, 710068, China; c State Key Laboratory for Mechanical Behavior of Materials, Shaanxi International Research Center for Soft Matter, School of Materials Science and Engineering, Xi’an Jiaotong University, Xi’an, 710049, China; d Rocket Force University of Engineering, Xi’an, 710025, China; e Frontier Institute of Science and Technology, Xi’an Jiaotong University, Xi’an, 710049, China; f Medical College, Xizang Minzu University, Xianyang, Shaanxi, 712082, China; g Institute of Medical Research, Northwestern Polytechnical University, Xi’an, 710072, China; h Institute of New Concept Sensors and Molecular Materials, Shaanxi Provincial Key Laboratory of New Concept Sensors and Molecular Materials, Xi’an Jiaotong University, Xi’an, 710049, China

## Abstract

• Three metallacycles (4a–4c) were constructed via multicomponent coordination-driven self-assembly as enhanced PDT platforms.
• Cationic metallacycles promote preferential accumulation in tumor cells, improving PDT efficacy.
• In vitro/in vivo assays showed potent cytotoxicity against HCT116/SW480 cells under light, with negligible dark toxicity.
• This self-assembly strategy enables precise integration of functional motifs, offering a promising supramolecular approach for PDT advancement.

## Graphical abstract

Novel TPE-based metallacycles were developed as efficient supramolecular photosensitizers, offering new opportunities for optimizing photodynamic therapy and advancing its biomedical applications.

**Figure:**
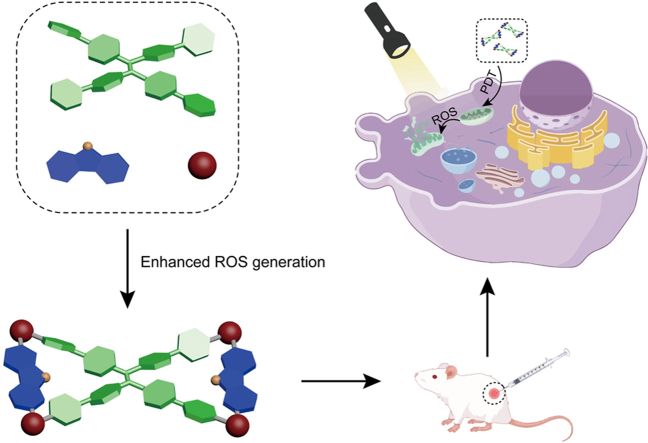

Cancer poses a severe threat to human health and life. Photodynamic therapy (PDT) has emerged as a highly promising tumor treatment modality due to its low toxicity and resistance to inducing drug resistance. Its core mechanism involves specific light sources exciting photosensitizers to generate reactive oxygen species (ROS), which subsequently kill tumor cells [1]. However, existing photosensitizers suffer from insufficient radical generation efficiency and limited functionality; to address this issue, this study designed three novel metal-based bicyclic photosensitizers through multicomponent coordination-driven self-assembled. The metal rings integrate the aggregation-induced emission (AIE) properties of tetraphenylethylene (TPE) with the electron-accepting capability of viologen derivatives. Concurrently, the incorporation of chalcogen atoms modulates electron coupling and conjugation effects, thereby enhancing electron transfer efficiency and photosensitization performance [2]. Furthermore, the cationic metal-ring structure promotes preferential accumulation within tumor cells, further improving the efficacy of photodynamic therapy [3,4]. *In vitro* and *in vivo* experiments confirm that under light irradiation, this series of compounds exhibits potent cytotoxic activity against human colorectal cancer cells. This photosensitizer design opens new avenues for developing highly efficient photosensitizers and optimizing photodynamic therapy.

Coordination-driven self-assembly technology provides a reliable and flexible tool for constructing multifunctional integrated supramolecular photosensitive platforms, enabling the precise and controllable integration of multiple functional modules into a single supramolecular structure. As shown in Fig. 1A, this study successfully synthesized three metal-ring compounds (**4a–4c**) using TPE ligand (**1**), dipyridyl ligands (**2a–2c**), and *cis*-Pt(PEt_3_)_2_(OTf)_2_ (**3**) as building blocks (See the Supplementary data for details). The resulting metallacycles were characterized by ^31^P{^1^H} nuclear magnetic resonance (NMR) and ^1^H NMR spectroscopy, and single-crystal X-ray diffraction (Fig. S1 and Table S1). In the absorption spectra, all three assemblies displayed intense π–π∗ transition bands spanning 250–350 nm, accompanied by a weaker broad feature centered around 450 nm (Fig. 1B). In the emission spectra, each metallacycle exhibits a maximum centered at approximately 510 nm, arising from the TPE core (Fig. 1C). A progressive decrease in fluorescence intensity from 4a to 4c was observed, in agreement with the incorporation of S, Se, and Te units that induce efficient quenching effects (Figs. S2–S4). These results demonstrate that the metallacycles emit in the blue–green spectral region with moderate efficiency, highlighting their potential as scaffolds for light-emitting system design.

**Fig. 1:**
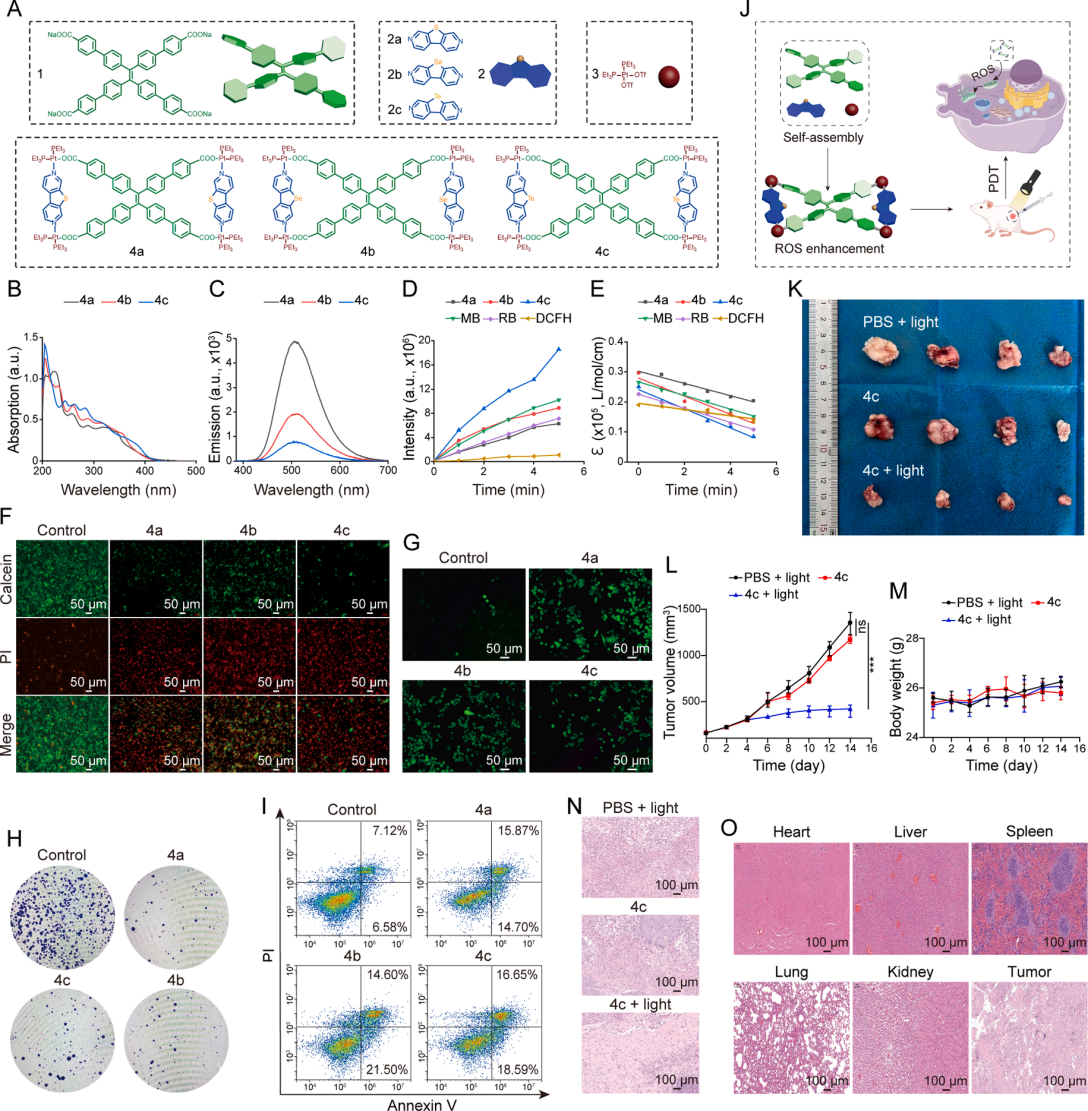
Characterization and photodynamic therapy (PDT) efficacy of multicomponent coordination-driven self-assembled metallacycles 4a–4c in colorectal cancer models. (A) Synthetic route of metallacycle compounds 4a–4c via multicomponent coordination-driven self-assembly. (B) Ultraviolet-visible spectrophotometry (UV-Vis) absorption spectra of metallacycles 4a–4c in acetonitrile. (C) Fluorescence emission spectra of metallacycles 4a–4c in acetonitrile. (D) Reactive oxygen species (ROS) generation efficiency of 4a–4c, methylene blue (MB) and rose bengal (RB) detected by 2',7'-dichlorofluorescein diacetate (DCFH-DA) probe under white light irradiation (50 mW/cm^2^, 0–5 min). (E) Singlet oxygen (^1^O_2_) generation efficiency of 4a–4c, MB and RB detected by 9,10-anthracenediyl-bis(methylene)dimalonic acid (ABDA) probe under white light irradiation. (F) Live/dead staining of HCT116 colorectal cancer cells after different treatments (green = live cells, red = dead cells). (G) Intracellular ROS detection of HCT116 cells after different treatments by DCFH-DA probe. (H) Colony formation assay of HCT116 cells after different treatments under light/dark conditions. (I) Apoptosis rate of HCT116 cells detected by flow cytometry 4 h after white light irradiation (50 mW/cm^2^, 10 min). (J) Schematic diagram of *in vivo* PDT treatment of colorectal cancer xenograft model in nude mice. Drawn by Figdraw. (K) Tumor photos of nude mice in each treatment group at the end of 14-day treatment (*n* = 4). (L) Tumor volume growth curves of nude mice in each group during 14-day treatment (*n* = 4, ^∗∗∗^*P* < 0.001, ns: not significant). (M) Body weight changes of nude mice in each group during the entire treatment cycle (*n* = 4). (N) Hematoxylin and eosin (H&E) staining of tumor tissues in each group. (O) H&E staining of major organs (heart, liver, spleen, lung, kidney) in 4c + light group.

The efficacy of photosensitizers is closely related to their ROS generation capacity. This study evaluated the photosensitizing activity of compounds 4a, 4b, and 4c by measuring their ROS generation efficiency. To validate the molecular design strategy employed in this research, the commercial photosensitizers methylene blue (MB) and rose bengal (RB) were selected as reference materials. Quantitative detection of total ROS production was performed using the fluorescent oxidative-sensitive probe 2',7'-dichlorofluorescein diacetate (DCFH-DA). Non-fluorescent DCFH-DA undergoes hydrolysis and is oxidized by ROS to form 2',7'-dichlorofluorescein (DCF), a strongly fluorescent compound emitting fluorescence at 525 nm. Experimental results showed that when white light exposure time was extended to 5 min, the DCF fluorescence intensity at 525 nm for compounds 4a, 4b, and 4c significantly increased. The fluorescence intensity increase in group 4c was significantly higher than in other groups. Group 4b was comparable to the MB group, while group 4a was comparable to the RB group, confirming that 4c possesses superior ROS generation capacity (Figs. 1D and S5. Regarding the role of singlet oxygen (^1^O_2_) in ROS generation, 9,10-anthracenediyl-bis(methylene) dimalonic acid (ABDA) was used as a probe to monitor ^1^O_2_ production (Fig. 1E). 4c exhibited superior ^1^O_2_ generation relative to MB and RB. Further singlet oxygen sensor green (SOSG) experiments and quantum yield determinations revealed that 4c possessed the fastest ^1^O_2_ generation rate and highest net fluorescence intensity, with a quantum yield of 0.86 (1.54-fold that of RB), exceeding 4b, 4a and RB (Fig. S6). These results were consistent with the ABDA assay.

The efficacy of PDT mediated by synthesized metallacycles was systematically evaluated *in vitro* using HCT116 colorectal cancer cells as a model. Cell Counting Kit-8 (CCK-8) assay results indicate that all compounds exhibit relatively low dark toxicity under light-protected conditions. Following phototherapy (50 mW/cm^2^, 10 min), cell viability exhibited a concentration-dependent decline. To investigate the mechanism of action, a sublethal concentration of 4 μM was selected for further study (Fig. S7). Live/dead staining assays experiments confirmed that, compared to the non-irradiated group (Fig. S8), the irradiated group (Fig. 1F) exhibited substantial cancer cell death. DCFH-DA probe assays verified that intracellular ROS generation was induced upon light irradiation, the core mechanism of PDT (Figs. 1G and S9). The colony formation assay results confirmed that the proliferation-inhibitory effects of these metallacycle compounds on HCT116 cells are light-dependent (Figs. 1H and S10). Flow cytometry analysis revealed that 4 h after light exposure, compounds 4a, 4b, and 4c induced apoptosis in HCT116 cells at rates of 30.57%, 36.1%, and 35.24%, respectively (Fig. 1I), significantly higher than the non-irradiated group (Fig. S11). This result confirms that the antitumor efficacy of this class of compounds originates from tumor cell apoptosis triggered by photodynamic therapy. All findings were validated in SW480 cells, confirming reproducibility and generalizability (Figs. S12–S16). HCT116 and SW480 human colorectal cancer cells were purchased from the Shanghai Institute of Biochemistry and Cell Biology, Chinese Academy of Sciences (SIBCB, CAS; Shanghai, China).

For *in vivo* PDT treatment, a well-established nude mouse xenograft model of colorectal cancer was employed, where nude mice bearing subcutaneous tumors with a volume of approximately 150 mm^3^ were selected as experimental subjects to ensure consistency in tumor progression stages (Fig. 1J). To ensure sufficient photosensitizer accumulation within tumor tissue, PDT was initiated 24 h post-injection. Subsequently, the tumor regions were irradiated with a laser (200 mW/cm^2^, 10 min). Treatment results showed that compound 4c alone only mildly inhibited tumor growth; however, the 4c plus light exposure group achieved significant tumor growth suppression (tumor volume nearly stagnated), with a statistically significant difference compared to the phosphate-buffered saline (PBS) control group (Figs. 1K and L). Tumor tissue weighing results across all groups further validated this conclusion (Fig. S17**)**. Importantly, no abnormal fluctuations in body weight were observed in any experimental mice throughout the entire treatment cycle (Fig. 1M). Hematoxylin and eosin (H&E) stained sections revealed significantly fewer cancer cells in the 4c + light group than in other groups **(**Fig. 1N**)**. Consistently, the 4c + light group showed a markedly lower percentage of Ki-67-positive cells and a higher proportion of terminal deoxynucleotidyl transferase-mediated dUTP nick-end labeling (TUNEL) positive cells relative to the PBS + light control, confirming that the photosensitizer exerts antitumor effects via inhibiting proliferation and inducing apoptosis (Figs. S18 and 19). Furthermore, H&E-stained sections of major organs showed no obvious morphological abnormalities (Fig. 1O and S20). Female BALB/c nude mice (4–5 weeks old) were purchased from Shaanxi Shaanyao Medical Biotechnology Co., Ltd. (Xi’an, China). All animal experiments were approved by the Ethics Committee of Xi’an Jiaotong University Health Science Center (Approval No.: XJTUAE2025-93) and conducted in accordance with the NIH Guide for the Care and Use of Laboratory Animals.

In summary, the TPE-based metal ring constructed through multicomponent coordination-driven self-assembly exhibits highly efficient ROS generation capability and demonstrates significant photocytotoxicity against cancer cells. The molecular design of this photosensitizer provides new directions for developing highly efficient photosensitizers and optimizing PDT.

## CRediT authorship contribution statement

**Jinping Zhang:** Writing – original draft, Validation, Project administration, Methodology, Formal analysis, Conceptualization. **Yali Hou:** Writing – review & editing, Methodology, Funding acquisition, Data curation. **Qian Feng:** Writing – review & editing, Methodology, Data curation. **Dengke Han:** Methodology, Data curation. **Qi Sun:** Software, Methodology. **Yuling Wu:** Methodology, Data curation. **Sida Liu:** Project administration, Methodology. **Dong Liu:** Funding acquisition. **Fei Xue:** Software, Data curation. **Jin Zhang:** Software, Data curation. **Jianing Wang:** Software, Data curation. **Xianglong Duan:** Writing – review & editing, Project administration, Funding acquisition. **Feng Liu:** Writing – review & editing, Project administration. **Mingming Zhang:** Writing – review & editing, Data curation, Conceptualization.

## Declaration of competing interest

The authors declare that they have no known competing financial interests or personal relationships that could have appeared to influence the work reported in this paper.

## References

[1] Cuadrado C.F., Lagos K.J., Stringasci M.D. (2024). Clinical and pre-clinical advances in the PDT/PTT strategy for diagnosis and treatment of cancer. Photodiagn. Photodyn. Ther..

[2] Zhou K., Tian R., Li G. (2019). Cationic chalcogenoviologen derivatives for photodynamic antimicrobial therapy and skin regeneration. Chem. Eur. J..

[3] Gong Q., Shi L., Wen L. (2025). Recent advances in metal ions overloading for tumors: Mechanisms, nanomaterials, and therapies. Mater. Today Bio..

[4] Yu G., Yu S., Saha M.L. (2018). A discrete organoplatinum(II) metallacage as a multimodality theranostic platform for cancer photochemotherapy. Nat. Commun..

