# Supplementary material for: Multicomponent coordination-driven self-assembled metallacycles as efficient photosensitizers for photodynamic therapy
Source: J Pharm Anal. 2026 Apr 27;16(8):101644. doi: 10.1016/j.jpha.2026.101644 (PMC13485630; doi:10.1016/j.jpha.2026.101644)
Supplement: Supplementary file 1 [file mmc1.docx]

**Supporting Information**

1. Materials and methods	2

2. Synthetic procedures and characterization data	6

Self-assembly of metallacycle 4a	6

Self-assembly of metallacycle 4b	8

Self-assembly of metallacycle 4c	10

3. Measurements of absolute fluorescence quantum yields for 4a ,4b and 4c	13

4. Assessment of Reactive Oxygen Species Yield for 4a, 4b, and 4c	14

5. In Vitro Photodynamic Therapy	15

6. In Vivo Photodynamic Therapy	19

Reference	21

## 1. Materials and methods

All reagents and deuterated solvents were purchased from Sigma-Aldrich (St. Louis, Missouri, USA) and used as purchased without further purification. TPE-based ligand 1 [S1] and dipyridyl ligands 2a–c [S2] were prepared according to the reported literature procedures. NMR spectra were recorded on a Bruker Avance 600 MHz spectrometer (Billerica, Massachusetts, USA). ^1^H NMR chemical shifts were recorded relative to residual solvent signals. ^31^P{^1^H} NMR chemical shifts were referenced to an external unlocked sample of 85% H_3_PO_4_ (*δ* 0.0). The UV-vis experiments were conducted on a Lambda 950 absorption spectrophotometer (PerkinElmer, Waltham, Massachusetts, USA). Fluorescence experiments were conducted on an Edinburgh FLS9 (Edinburgh Instruments, Livingston, UK) or a Hitachi F-7000 (Tokyo, Japan) fluorescence spectrophotometer. X-ray diffraction analysis was conducted on a Bruker D8 VENTURE PHOTON II MetalJet diffractometer (Billerica, Massachusetts, USA)，in which crystals were frozen in paratone oil diffractometer inside a cryoloop under a cold stream of N_2_. An empirical absorption correction using SADABS was applied for all data. The structures were solved and refined to convergence on F2 for all independent reflections by the full-matrix least squares method using the OLEX2 1.2 software (Olex2 Ltd, Manchester, UK).

**Reactive oxygen species**

DCFH-DA (4.87 mg, Sigma-Aldrich, St. Louis, Missouri, USA) was dissolved in DMSO (1.0 mL，Sigma-Aldrich, St. Louis, Missouri, USA) to afford a 10 mM stock solution. An aliquot (200 μL) of this stock was diluted with DMSO (1.8 mL) to prepare a 1.0 mM DCFH-DA solution. The solution was treated with NaOH (Sinopharm Chemical Reagent Co., Ltd, Shanghai, China) (8.0 mL, 0.01 M) and kept in the dark at room temperature for 30 min. The resulting mixture was neutralized by the addition of PBS (90 mL，Thermo Fisher Scientific, Waltham, MA, USA) to afford a 2.0 μM DCFH solution. This solution (2.0 mL) was mixed with 1.0 μL of 2.0 mM 4a, 4b, or 4c, respectively, and the mixtures were irradiated with white light (50 mW cm⁻²). MB (Sigma-Aldrich, St. Louis, Missouri, USA) and RB (Sigma-Aldrich, St. Louis, Missouri, USA) were used as positive controls, both at the same concentration as the metallacycle samples (4a–4c). The fluorescence intensity at 525 nm (*λ*_ex_ = 488 nm) was recorded to quantify ROS generation.

**Singlet oxygen measurements**

The method for determining the singlet oxygen quantum yield was based on a previously reported literature procedure[S3]. ABDA (Sigma-Aldrich, St. Louis, Missouri, USA) was used as a specific probe for singlet oxygen trapping. Specifically, a 5 mM ABDA stock solution and a 1 mM sample solution were prepared. The reaction mixture was formulated by mixing 2 mL of ultrapure water, 18 μL of ABDA stock solution, and 2 μL of the sample solution. After the sample was irradiated with ultrasound (MEJIRO GENOSSEN, Tokyo, Japan, power density: 50 mW/cm²), UV-visible absorption spectra were recorded at 1.0-minute intervals, and the absorbance values of ABDA at 400 nm were documented. The quantum yield of singlet oxygen generation was calculated based on the slope of the absorbance-time change curve.

**Cell culture**

Human colon cancer cell lines HCT116 and SW480 were purchased from the Shanghai Institute of Cell Biology in the Chinese Academy of Sciences. HCT116 cells were cultured in Roswell Park Memorial Institute 1640 medium (RPMI 1640, Gibco, Grand Island, New York, USA) supplemented with 10% fetal bovine serum (FBS , Gibco, Grand Island, New York, USA) and antibiotics (100 IU/mL penicillin and 100 µg/mL streptomycin，Wuhan, Hubei, China), and SW480 was cultured in Dulbecco's modified Eagle's medium (DMEM, Gibco, Grand Island, New York, USA) supplemented with the same FBS and antibiotics. Both cell lines were incubated in a humidified atmosphere of 5% CO2 at 37°C (Shanghai, China).

**Cell viability assay**

Cells were seeded into 96-well plates (Corning Inc., Corning, New York, USA) at a density of 5×10^3^cells per well in 200 µL of complete culture medium and incubated in a humidified atmosphere of 5% CO₂ at 37°C for 24 h. The culture medium was then aspirated and replaced with freshly prepared medium containing different synthetic compounds at various concentrations. The plates were subsequently exposed to 50mW/cm² white light (400-760 nm，10 min) or maintained in the dark for the same duration. After an additional 24 h of incubation, the medium was replaced with fresh culture medium, and 10 µL of CCK8 (Shanghai, China) solution was added to each well. The plates were incubated at 37°C for 1 h, after which the absorbance was measured at 450 nm using a Bio-Rad 450 microplate reader (Bio-Rad Laboratories, Hercules, California, USA). The IC₅₀ values were calculated using GraphPad Prism software (Version 9.0) based on data from five independent experiments.

**Colony Formation Assay**

Each cell type was seeded into the center of a 6-well plate (Corning Inc., Corning, New York, USA) (1,000 cells per well) and cultured for 24 hours at 37°C in a 5% CO₂ incubator. The medium was then aspirated and replaced with freshly prepared medium containing 4 µM synthetic compound. The culture plates were exposed to 50 mW/cm² white light for 10 minutes or incubated in the dark for 10 minutes. After 24 h of continuous culture, fresh medium was replenished, and the cells were further cultured for another 24 h. This process was repeated. HCT116 cells were cultured for 14 days, and SW480 cells for 21 days. Following medium removal, cells were fixed with 4% formaldehyde solution (Shanghai, China) for 10 minutes, and the formaldehyde solution was discarded. Cells were stained with 0.1% crystal violet (Shanghai, China) for 10 minutes, washed with water, and then subjected to colony counting.

**Flow Cytometric Apoptosis Assay**

Seed 5×10⁵ cells into a 6-well plate and add corresponding samples or PBS. Culture cells under white light irradiation (power density: 50 mW/cm², 10 min) or in dark conditions. After 4 hours, digest cells with trypsin (Gibco, Grand Island, New York, USA), gently resuspend in PBS, and collect cell pellets by centrifugation at 1000×g for 5 min. Subsequently, add 195 μL Annexin V-FITC binding buffer (Beyotime Biotechnology Co., Ltd, Shanghai, China) to the pellet, followed by 5 μL Annexin V-FITC. Gently mix. Then add 10 μL propidium iodide (PI) staining solution (Beyotime Biotechnology Co., Ltd, Shanghai, China) and gently mix again. Incubate cells at room temperature (20-25°C) in the dark for 10-20 minutes, then immediately proceed to flow cytometry analysis (BD Biosciences, Franklin Lakes, New Jersey, USA).

**Live/Dead Cell-Containing Assays**

HCT116 and SW480 cells were seeded at a density of 2 × 10⁴ cells per well in 24-well plates and cultured for 24 hours under standard cell culture conditions (37°C, 5% CO₂). Fresh medium was then replaced with either 4 μM compound or PBS. The mixture was exposed to 50 mW/cm² white light for 10 minutes or incubated in the dark for 10 minutes. After 12 hours of further culture, cells were stained with 500 μL of calcein AM/propidium iodide dye (Beyotime Biotechnology Co., Ltd, Shanghai, China) for 25 minutes, followed by observation of green (492 nm) and red (545 nm) fluorescence under a fluorescence microscope (Optec Instrument Co., Ltd, Chongqing, China).

**In vitro** **reactive oxygen species (ROS) measurements**

Cells were seeded at a density of 5 × 10⁴ cells per well in a 24-well plate and cultured to the logarithmic growth phase. After replacing the medium with freshly prepared medium containing 4 µM synthetic compound, the culture plate was exposed to 50 mW/cm² white light for 10 minutes or incubated in darkness for the same duration. After 24 hours of continuous culture, media was removed and cells were washed twice with PBS. A 10 µM DCFH-DA (Beyotime Biotechnology Co., Ltd, Shanghai, China) working solution diluted in serum-free medium was added, and cells were incubated in the dark at 37°C with 5% CO₂ for 20–30 minutes. Wash cells three times with serum-free medium to remove extracellular free DCFH-DA, then acquire fluorescence images using 488 nm excitation light.

**In vivo tumor therapy study**

The Ethics Committee of Xi'an Jiaotong University Health Science Center (Xi’an, China) approved all animal experiments (Approval No.: XJTUAE2025-93), which followed the NIH-Guide for the Care and Use of Laboratory Animals. To evaluate the PDT efficiency of the synthetic compound 4c, its antitumor performance was validated in vivo via intratumoral administration. The intratumoral injection volume was 100 µL, the solute in the injection preparation was photosensitizer, and the mixed solvent was 99% PBS and 1% DMSO. Six-week-old BALB/c-nu mice were subcutaneously inoculated with a 1:1 mixture of HCT116 cell suspension and Matrigel at a final concentration of 1×10^6^ cells/100 µL. Using a 27G needle, the cell mixture was slowly injected subcutaneously at a 30° angle to the skin. Treatment was initiated when tumor volume reached approximately 150 mm³ (∼1week post-inoculation). A total of 12 tumor-bearing mice were randomly assigned to three groups (n=4 per group), with the following interventions: Group I (PBS + Light): Intratumoral injection of PBS (100 µL) followed by light irradiation (450 nm, 200mW/cm², 10 min). Group II (4c): Intratumoral administration of 4c (10 mg/mL, 50 mg/kg) via four-quadrant injections into the tumor (based on nude mouse body weight). Group III (4c + Light): Intratumoral injection of 4c (10 mg/mL, 50 mg/kg) followed by light irradiation (450 nm, 200mW/cm², 10 min). The treatment frequency was once every 2 days. Tumor volumes were measured every 2 days using a vernier caliper during the treatment period, Tumor volume *V* = length × width^2^/2. After 14 days of treatment, tumors were harvested from all groups and weighed.

**Statistical Analysis**

All measured values in this study are presented as mean ± SEM or mean ± SD, and data analysis was performed using GraphPad Prism 9.0 software. All experiments were independently repeated three times, and the results represent the pooled data from these independent experiments. Statistical significance was assessed by two-way ANOVA, one-way ANOVA （or a mixed model). The significance levels were defined as follows: * *P* < 0.05 (significant difference), ** *P* < 0.01 (highly significant difference), *** *P* < 0.001 (very highly significant difference), and **** *P* < 0.0001 (extremely significant difference). *P* < 0.05 was considered statistically significant in all analyses.

## 2. Synthetic procedures and characterization data

**Self-assembly of metallacycle 4a**

**1** (3.0 mg，3.33 µmol), **2a** (1.24 mg，6.66 µmol) and **3** (9.72 mg，13.32 µmol) were mixed in a 1:2:4 molar ratio and dissolved in acetone/water (20 mL, 4:1, *v*/*v*). The reaction mixture was heated at 50 ̊C for 12 hours and then cooled to room temperature. The solvent was removed by nitrogen flow. The residue was redissolved in CH_3_CN (1.0 mL), filtered, and treated with ethyl ether (4 mL) to induce precipitation. The resulting precipitate was collected via centrifugation, yielding metallacycle **4a** (9.20 mg, 95%) as a yellow solid. ^1^H NMR (600 MHz, CD_3_CN) δ 9.58 (d, J = 2.7 Hz, 4H), 8.95 (dd, J = 6.0, 2.2 Hz, 4H), 8.44 (d, J = 6.2 Hz, 4H), 7.68 (d, J = 8.4 Hz, 8H), 7.38 (d, J = 8.3 Hz, 8H), 7.24 (d, J = 8.3 Hz, 8H), 7.03 (d, J = 8.2 Hz, 8H).^31^P{^1^H} NMR (243 MHz, CD_3_CN, 295 K): 5.76 ppm (d, ^2^*J*_P-P_ = 20.7 Hz, ^195^Pt satellites, *^1^J*_Pt-P_ = 1674.3 Hz), –0.08 ppm (d, ^2^*J*_P-P_ = 20.7 Hz, ^195^Pt satellites, *^1^J*_Pt-P_ = 1674.3 Hz).

^1^H NMR spectrum (600 MHz, CD_3_CN, 295 K) recorded for **4a**.

^31^P{^1^H} NMR spectrum (243 MHz, CD_3_CN, 295 K) recorded for **4a**.

**Self-assembly of** **metallacycle 4b**

**1** (4.0 mg, 4.4 µmol), **2b** (2.07 mg, 8.9 µmol) and **3** (13.0 mg,17.8 µmol) were mixed in a 1:2:4 molar ratio and dissolved in acetone/water (20 mL, 4:1, *v*/*v*). The whole reaction mixture was heated at 50 ̊C for 12 hours and then cooled to room temperature. The solvent was removed by nitrogen flow. The residue was redissolved in CH_3_CN (1.0 mL), filtered, and treated with ethyl ether (4 mL) to induce precipitation. The resulting solid was collected by centrifugation to afford metallacycle **4b** (12.52 mg, 94%) as a yellow solid. ^1^H NMR (600 MHz, CD_3_CN) δ 9.55 (d, J = 2.9 Hz, 4H), 8.93 (dd, J = 5.9, 2.5 Hz, 4H), 8.37 (d, J = 6.2 Hz, 4H), 7.68 (d, J = 8.4 Hz, 8H), 7.39 (d, J = 8.4 Hz, 8H), 7.25 (d, J = 8.3 Hz, 8H), 7.03 (d, J = 8.3 Hz, 8H).^31^P{^1^H} NMR (243 MHz, CD_3_CN, 295 K): 5.69 ppm (d, ^2^*J*_P-P_ =72.9 Hz, ^195^Pt satellites, *^1^J*_Pt-P_ =1687.05 Hz), 0.07 ppm (d, ^2^*J*_P-P_ =22.7 Hz, ^195^Pt satellites, *^1^J*_Pt-P_ = 1687.05 Hz).

^1^H NMR spectrum (600 MHz, CD_3_CN, 295 K) recorded for **4b**.

^31^P{^1^H} NMR spectrum (243 MHz, CD_3_CN, 295 K) recorded for **4b**.

**Self-assembly of metallacycle 4c**

**1** (3.0 mg, 3.3 µmol), **2c** (1.9 mg, 6.7 µmol) and **3** (9.7 mg, 13.3 µmol) were mixed in a 1:2:4 molar ratio and dissolved in acetone/water (20 mL, 4:1, *v*/*v*). The whole reaction mixture was heated at 50 ̊C for 12 hours and then cooled to room temperature. The solvent was removed by nitrogen flow. The residue was redissolved in CH_3_CN (1.0 mL), filtered, and treated with ethyl ether (4 mL) to induce precipitation. The resulting solid was collected by centrifugation to afford metallacycle **4c** (12.8 mg, 96%) as a yellow solid. ^1^H NMR (600 MHz, CD_3_CN) δ 9.49 (d, *J* = 2.5 Hz, 4H), 8.85 (dd, *J* = 6.1, 2.2 Hz, 4H), 8.30 (d, *J* = 6.2 Hz, 4H), 7.68 (d, *J* = 8.2 Hz, 8H), 7.40 (d, *J* = 8.3 Hz, 8H), 7.26 (d, *J* = 8.2 Hz, 8H), 7.04 (d, *J* = 8.2 Hz, 8H). ^31^P{^1^H} NMR (243 MHz, CD_3_CN, 295 K): 5.61 ppm (d, ^2^*J*_P-P_ =19.5 Hz, ^195^Pt satellites, *^1^J*_Pt-P_ =1687.05 Hz), 0.05 ppm (d, ^2^*J*_P-P_ =19.5 Hz, ^195^Pt satellites, *^1^J*_Pt-P_ = 1687.05 Hz).

^1^H NMR spectrum (600 MHz, CD_3_CN, 295 K) recorded for **4c**.

^31^P{^1^H} NMR spectrum (243 MHz, CD_3_CN, 295 K) recorded for **4c**.

**Fig S1.** Partial ¹H nuclear magnetic resonance (NMR) spectra (400 MHz, CD₃CN, 295 K) of **2a** (a), **4a** (b), **2b** (c), **4b** (d), **2c** (e), and **4c** (f). Partial ³¹P{¹H} NMR spectra (121.4 MHz, CD₃CN, 295 K) of **4a** (g), **4b** (h), and **4c** (i). Single-crystal structure of **4b** (j). crystal packing model of **4b** (k). Hydrogen atoms, counterions, solvent molecules, and triethylphosphine units are omitted for clarity.

**Table S1.** Crystallographic data and refinement details for metallacycle **4b**

| Compound | **4b** |
| --- | --- |
| Empirical formula | C_274_ H_368_ F_6_ N_12_ O_30_ P_16_ Pt_8_ |
| Fw | 6544.14 |
| Crystal system | triclinic |
| Space group | *P-1* |
| *a* /Å | 17.6234 |
| *b* /Å | 17.8682 |
| *c* /Å | 27.0579 |
| *α* /° | 89.588 |
| *β* /° | 83.413 |
| *γ* /° | 73.118 |
| *V* /Å^3^ | 8096.42 |
| Z | 2 |

## 3. Measurements of absolute fluorescence quantum yields for 4a ,4b and 4c

**Fig S2.** Absolute fluorescence quantum yield of metallacycle **4a** in CH_3_CN.

**Fig S3.** Absolute fluorescence quantum yield of metallacycle **4b** in CH_3_CN.

**Fig S4.** Absolute fluorescence quantum yield of metallacycle **4c** in CH_3_CN.

## 4. Assessment of Reactive Oxygen Species Yield for 4a, 4b, and 4c

**Fig S5.** Plots of relative photoluminescence (PL) intensity at 525 nm of 2',7'-dichlorofluorescein diacetate (DCFH-DA) for total Reactive Oxygen Species (ROS) detection under white light irradiation (50 mW/cm², 0–5 min). Data are presented as mean ± SD (n = 3).

**Fig S6.** Plots of relative PL intensity of singlet oxygen sensor green (SOSG) for Singlet oxygen (¹O₂) detection under white light irradiation (50 mW/cm², 0–5 min). Data are presented as mean ± SD (n = 3).

## 5. In Vitro Photodynamic Therapy

**Fig S7.** Cell viability of HCT116 cells treated with different concentrations of **4a**, **4b**, and **4c** in the presence and absence of light. Data are collected and shown as the mean ± SD (*n* = 3), ***P* < 0.01.*****P* < 0.001.

**Fig S8.** Live/dead cell co-staining assays using calcein-AM and propidium iodide (PI) as fluorescent probes, after incubation with **4a**, **4b**, and **4c** followed by non-irradiated (scale bars = 50 μm). Green = live cells, red = dead cells.

**Fig S9.** Intracellular ROS imaging of HCT116 cells after incubation with 4a, 4b, and 4c under non-irradiated conditions (DCFH-DA probe, λₑₓ = 488 nm). Scale bars = 50 μm.

**Fig S10.** Assessment of HCT116 Cell Colony Formation Capacity. After treatment with 4a, 4b, and 4c, cells were cultured in the dark for 14 days and stained with crystal violet.

**Fig S11.** Apoptosis analysis of HCT116 cells treated with 4a, 4b, and 4c for 4 hours under non-irradiated conditions (Annexin-V/PI staining, flow cytometry). Data are presented as mean ± SD (n = 3).

**Fig S12.** Cell viability of SW480 cells treated with different concentrations of 4a, 4b, and 4c in the presence and absence of white light irradiation (50 mW/cm², 10 min). Data are presented as mean ± SD (n = 3), ****P < 0.0001 vs. the dark group.

**Fig S13.** Live/dead cell co-staining assays of SW480 cells using calcein-AM and PI as fluorescent probes after incubation with 4a, 4b, and 4c under white light irradiation (50 mW/cm², 10 min). Scale bars = 50 μm. Green = live cells, red = dead cells.

**Fig S14.** Intracellular ROS imaging of SW480 cells after incubation with 4a, 4b, and 4c in the absence and presence of white light irradiation (DCFH-DA probe, λₑₓ = 488 nm). Scale bars = 50 μm.

**Fig S15.** Colony formation assay of SW480 cells after treatment with 4a, 4b, and 4c under light/dark conditions for 21 days (crystal violet staining).

**Fig S16.** Apoptosis analysis of SW480 cells treated with 4a, 4b, and 4c for 4 hours in the presence and absence of white light irradiation (Annexin-V/PI staining, flow cytometry). Data are presented as mean ± SD (n = 3).

## 6. In Vivo Photodynamic Therapy

**Fig S17.** Subcutaneous tumor weight of nude mice in different treatment groups after 14 days of PDT treatment. Data are presented as mean ± SEM (n = 4), ***P < 0.001 vs. PBS + Light group and 4c alone group.

**Fig S18.** Ki67 immunohistochemical staining of tumor tissues from nude mice after 14 days of PDT treatment. Scale bars = 100 μm. Ki67-positive cells (brown) indicate proliferating tumor cells; nuclei were counterstained with hematoxylin (blue).

**Fig S19*.*** Terminal Deoxynucleotidyl Transferase-Mediated dUTP Nick-End Labeling (TUNEL) staining of tumor tissues after PDT treatment (scale bars = 100 μm). TUNEL-positive cells (green/red, apoptotic cells); nuclei were counterstained with DAPI (blue).

**Fig S20*.*** Hematoxylin and eosin (H&E) staining of major organs (heart, liver, spleen, lung, kidney) from nude mice in the 4c + Light group after 14 days of PDT treatment. Scale bars = 100 μm. No obvious morphological abnormalities or tissue damage were observed).

## Reference

[S1] Z. Zhang, Z. Zhao, Y. Hou, H. Wang, X. Li, G. He, M. Zhang, Aqueous Platinum (II)-Cage-Based Light-Harvesting System for Photocatalytic Cross-Coupling Hydrogen Evolution Reaction，*Angew. Chem. Int. Ed.* **2019**, *58*, 8862.

[S2] G. Li, B. Zhang, J. Wang, H. Zhao, W. Ma, L. Xu, W. Zhang, K. Zhou, Y. Du, G. He, Electrochromic Poly(chalcogenoviologen)s as Anode Materials for High-Performance Organic Radical Lithium-Ion Batteries， *Angew. Chem. Int. Ed.* **2019**, *58*, 8468.

[S3] Zhu C, Yang Q, Liu L, et al. Zhu, C., Yang, Q., Liu, L., Lv, F., Li, S., Yang, G. and Wang, S. (**2011**), Multifunctional Cationic Poly (p-phenylene vinylene) Polyelectrolytes for Selective Recognition, Imaging, and Killing of Bacteria Over Mammalian Cells. *Adv. Mater.*, 23: 4805-4810.
